# Gene Expression Signature in Endemic Osteoarthritis by Microarray Analysis

**DOI:** 10.3390/ijms160511465

**Published:** 2015-05-19

**Authors:** Xi Wang, Yujie Ning, Feng Zhang, Fangfang Yu, Wuhong Tan, Yanxia Lei, Cuiyan Wu, Jingjing Zheng, Sen Wang, Hanjie Yu, Zheng Li, Mikko J. Lammi, Xiong Guo

**Affiliations:** 1 School of Public Health, Xi’an Jiaotong University Health Science Center, Key Laboratory of Trace Elements and Endemic Diseases, National Health and Family Planning Commission, No. 76 Yanta West Road, Xi’an 710061, China; E-Mails: wx231115210@stu.xjtu.edu.cn (X.W.); yujie.ning@sydney.edu.au (Y.N.); fzhxjtu@mail.xjtu.edu.cn (F.Z.); y844481760@stu.xjtu.edu.cn (F.Y.); tanwh@mail.xjtu.edu.cn (W.T.); leiyanx@163.com (Y.L.); xj.cy.69@stu.xjtu.edu.cn (C.W.); zhengjingjing.1984@163.com (J.Z.); alison@stu.xjtu.edu.cn (S.W.); 2 National Engineering Research Center for Miniaturized Detection Systems, Northwest University, Xi’an 710069, China; E-Mails: yuhanjie0205@163.com (H.Y.); zhengli@nwu.edu.cn (Z.L.); 3 Department of Integrative Medical Biology, University of Umea, 901 87 Umeå, Sweden; E-Mail: mikko.lammi@umu.se

**Keywords:** Kashin-Beck disease, microarray, peripheral blood mononuclear cells, gene expression signature

## Abstract

Kashin-Beck Disease (KBD) is an endemic osteochondropathy with an unknown pathogenesis. Diagnosis of KBD is effective only in advanced cases, which eliminates the possibility of early treatment and leads to an inevitable exacerbation of symptoms. Therefore, we aim to identify an accurate blood-based gene signature for the detection of KBD. Previously published gene expression profile data on cartilage and peripheral blood mononuclear cells (PBMCs) from adults with KBD were compared to select potential target genes. Microarray analysis was conducted to evaluate the expression of the target genes in a cohort of 100 KBD patients and 100 healthy controls. A gene expression signature was identified using a training set, which was subsequently validated using an independent test set with a minimum redundancy maximum relevance (mRMR) algorithm and support vector machine (SVM) algorithm. Fifty unique genes were differentially expressed between KBD patients and healthy controls. A 20-gene signature was identified that distinguished between KBD patients and controls with 90% accuracy, 85% sensitivity, and 95% specificity. This study identified a 20-gene signature that accurately distinguishes between patients with KBD and controls using peripheral blood samples. These results promote the further development of blood-based genetic biomarkers for detection of KBD.

## 1. Introduction

Kashin-Beck disease (KBD) is an endemic osteochondropathy that manifests with significant alterations in chondrocyte phenotype, necrosis, apoptosis, and abnormal terminal chondrocyte differentiation [1]. The disease occurrence is mainly distributed in a diagonal belt ranging from the northeast to the southwest of China, which is a region with low selenium content in the soil. Over 60,000 patients are affected by the disease and approximately 30 million residents are at risk [2], which most of them living in the remote rural or mountainous areas with poor transportation, low incomes, and poor diets. The etiology and pathogenesis of KBD are still unclear, although two etiologic hypotheses for KBD have been proposed: (1) selenium deficiency in the internal and external environments of endemic KBD areas and (2) serious cereal contamination by mycotoxins produced by fungi [3,4].

Currently, the diagnosis and determination of the severity of KBD is primarily based on the symptoms, such as joint pain, enlargement of phalanges or other joints, limitation of motion, arthralgia, deformity of limbs and metaphysis changes observed radiologically. However, this is effective only for diagnosis of advanced cases, which eliminates the possibility of early treatment and lead to inevitable exacerbation and irreversibility of symptoms like deformity of limbs and dwarfism. The treatments of KBD, which include symptomatic treatment, articular injection of sodium hyaluronate to relieve the pain and symptoms, and arthroplasty [5] to replace the damaged cartilage with artificial joints, are the only effective measures to treat the patients. Therefore, early diagnosis plays a critical role in the therapy of KBD, although several problems exist: (1) clinical signs and symptoms become obvious only after joint degeneration has occurred; and (2) the progression of cartilage injury and degradation is slow, making it difficult to detect the disease in the early stage.

Blood is considered to be an ideal marker for disease diagnosis, as it is relatively easy to collect without any obvious trauma. Due to the interactions and communication between tissues and organs, blood can accurately reflect the current state of health or disease of the human body. Ma and Liew demonstrated that blood-derived RNA expression could be used for disease diagnosis [6], and numerous subsequent studies have verified these findings using microarrays to diagnose a series of diseases including osteoarthritis (OA) [7]. Several recent studies of OA, of KBD and in other fields suggest that systems biology may play an important role in the understanding of the etiology of the diseases and pathophysiological processes [1,8,9,10,11]. The dynamic and interactive properties of blood can be effectively applied to osteoarthropathy, such as KBD.

Therefore, we focused on the peripheral blood of patients with KBD to identify a specific gene signature that could be associated with the early diagnosis and the detection of KBD. In this study, 169 genes were selected as target genes, based on the results of previous genome-wide gene expression profile analysis in articular cartilage and peripheral blood mononuclear cells (PBMCs) from KBD patients [9,12] The expression levels of the 169 target genes in peripheral blood of 100 KBD patients and 100 healthy controls were evaluated via a microarray analysis. Based on the results, a 20-gene signature was identified and validated as a highly sensitive and specific tool for detecting KBD in the peripheral blood.

## 2. Results

### 2.1. Differentially Expressed Genes

The microarray system was used to compare the gene expression levels of 169 target genes in the PBMCs from KBD patients *versus* controls. Fifty genes were identified as differentially expressed (18 up-regulated and 32 down-regulated) in the 100 paired of microarray data sets.

### 2.2. Identification of a 20-Gene Signature

The training set (*n =* 160) was divided into an inner training set (*n =* 159) and an inner test set (*n =* 1). The mRMR algorithm was used to select a subset of n genes which exhibited a maximum relevance to the clinical status of the KBD and also a minimum redundancy within the 50 differentially expressed genes identified by microarray analyses. Next, the SVM model was built in the inner training set by using the linear kernel based on *n* genes selected by mRMR. Two parameters of SVM, which determined the accuracy of the model, were optimized by the program named gridergression.py. First, the parameters “c” abbreviate from “cost”: set the parameter C of C-SVC, epsilon-SVR, and nu-SVR (default 0.5); second, the parameters “g” abbreviate from “gamma”: set gamma in kernel function (default 1/k). Then, the SVM model was used to predict the class of the inner test set. The prediction was dependent on the expression level of the inner test set without knowledge of the true class of the test sample. We compared the predicted class of the sample with the true class label of the inner test set. If they were consistent, the prediction was correct.

Then, a new classification model was built with the new inner training set and the inner test set. This time, another sample was in the inner test set, and all of the other 159 samples were in the inner training set. With the same mRMR algorithm for gene selection, the new SVM model based on the new training set was built, and the gene set selection was not exactly the same as in the previous model. Once again, the SVM model was used to predict the class of the inner test set. If the prediction was consistent with the true class label, then the prediction was correct. The process described above was repeated by leaving each of the 160 samples out of the training set one at a time. Therefore, 160 different SVM models were built, and each model was performed to predict the class of the inner test set. Finally, the number of the correct predictions was summed and considered the LOOCV (leave-one-out cross validation) accuracy rate. The amount of genes selected by the mRMR algorithm could be set as a predefined variable. We began with one gene, and added one gene at a time to *n =* 50. Then, we calculated the accuracy rate of LOOCV for each *n* genes and the optimal gene signature was determined by LOOCV performance. Eventually, 8000 different SVM models were built to evaluate the gene signature performance. The classification accuracy was 97.5% when 20 genes were included in the signature, and the accuracy increased when more genes were added to the signature.

Our aim in this study was to identify a high-performing gene signature with compact size, which would be a rational application for future development. Therefore, we identified a 20-gene signature as the optimal size. Finally, the 20-gene signature identified by the mRMR algorithm and the SVM model in the training set was used to discriminate between patients with KBD and the normal controls in the test set (*n =* 40). The prediction was based on the expression level of the test set without using knowledge of the true KBD class of the sample. The twenty gene expression signature discriminated the KBD patients from the controls with 90% accuracy, 85% sensitivity, and 95% specificity.

### 2.3. Statistical Analysis of the KBD Degree with the 20-Gene Signature

To further elucidate the relationship between signature genes and detection of KBD, Bayes discriminant analysis (BDA) algorithm was performed with the 20-gene signature (Table 1) to determine the KBD degree. Based on the expression ratios of the 20 signature genes, BDA correctly classified the KBD degree (I, II) in 91% (91/100) of the patients. After a LOOCV, BDA correctly classified the KBD degree in 82% (82/100) of the KBD patients (Table 2). In addition, linear correlation analyses showed that none of the 20 genes was found to be differentially expressed based on age. When 100 KBD patients were divided into four age groups and an ANOVA test was performed to observe the interaction between age and gene expression, there were no significant differences in gene expression among the four age groups.

**Table 1:** List of the 20 signature genes in KBD patients ^a^.

Gene Name	Symbol	Public ID	Fold Change ^b^
***down-regulated genes***			
ATP-binding cassette, sub-family C, member 13, pseudogene	ABCC13	NR_003087	0.42 ± 0.03
ABI family, member 3 (NESH) binding protein	ABI3BP	NM_015429	0.33 ± 0.03
Branched chain amino-acid transaminase 1, cytosolic	BCAT1	NM_001178091	0.43 ± 0.04
Calcium channel, voltage-dependent, gamma subunit 6	CACNG6	NM_145814	0.39 ± 0.02
Chondroitin sulfate *N*-acetylgalactosaminyltransferase 1	CSGALNACT1	NM_001130518	0.21 ± 0.03
Cathepsin C	CTSC	NM_001114173	0.39 ± 0.02
Cytochrome b5 reductase 3	CYB5R3	NM_000398	0.48 ± 0.04
Dystrophin, muscular dystrophy	DMD	NM_007868	0.37 ± 0.02
Enhancer of rudimentary homolog (Drosophila)	ERH	NM_004450	0.38 ± 0.02
F11 receptor	F11R	NM_016946	0.46 ± 0.02
FK506 binding protein 9, 63 kDa	FKBP9	NM_007270	0.49 ± 0.06
Frizzled family receptor 1	FZD1	NM_003505	0.47 ± 0.03
Growth differentiation factor 5	GDF5	NM_000557	0.44 ± 0.03
Hemoglobin, alpha 2	HBA2	NM_000517	0.49 ± 0.03
Zinc family member 5	ZIC5	NM_033132	0.38 ± 0.02
***up-regulated genes***			
Baculoviral IAP repeat containing 3	BIRC3	NM_001165	4.26 ± 0.35
FGFR1 oncogene partner 2	FGFR1OP2	NM_001171887	2.39 ± 0.15
Sialic acid binding lg-like lectin 8	SIGLEC8	NM_014442	2.50 ± 0.29
Single-stranded DNA binding protein 1, mitochondrial	SSBP1	NM_001256510	3.12 ± 0.25
Tetratricopeptide repeat domain 25	TTC25	NM_031421	3.19 ± 0.25

^a^ Differentially expressed genes between the KBD *vs.* controls were assessed using the selection criteria described in Materials and Methods. According to the fold change value, only those genes that showed significant differences (*p*-value < 0.05) in expression were screened as differential expression genes in KBD and were identified as signature genes are listed; ^b^ fold change, the mean and standard error of the mean (SEM) of the fold change in expression of each gene.

**Table 2:** Results of Bayes discriminant analysis (BDA) with the expression ratio of signature genes ^b,c^.

Diagnosed Group Membership			Predicted Group Membership		Total
			KBD Degree I	KBD Degree II	
Original	Count	Degree I	46	4	50
		Degree II	5	45	50
	%	Degree I	92.0	8.0	100.0
		Degree II	10.0	90.0	100.0
Cross-validated ^a^	Count	Degree I	41	9	50
		Degree II	9	41	50
	%	Degree I	82.0	18.0	100.0
		Degree II	18.0	82.0	100.0

^a^ Cross validation is performed only for those cases in the analysis. In cross validation, each case is classified by the functions derived from all cases other than that case; ^b^ 91.0% of original grouped cases correctly classified; ^c^ 82.0% of cross-validated grouped cases correctly classified.

### 2.4. Quantitative RT-PCR Analyses

**Figure 1:**
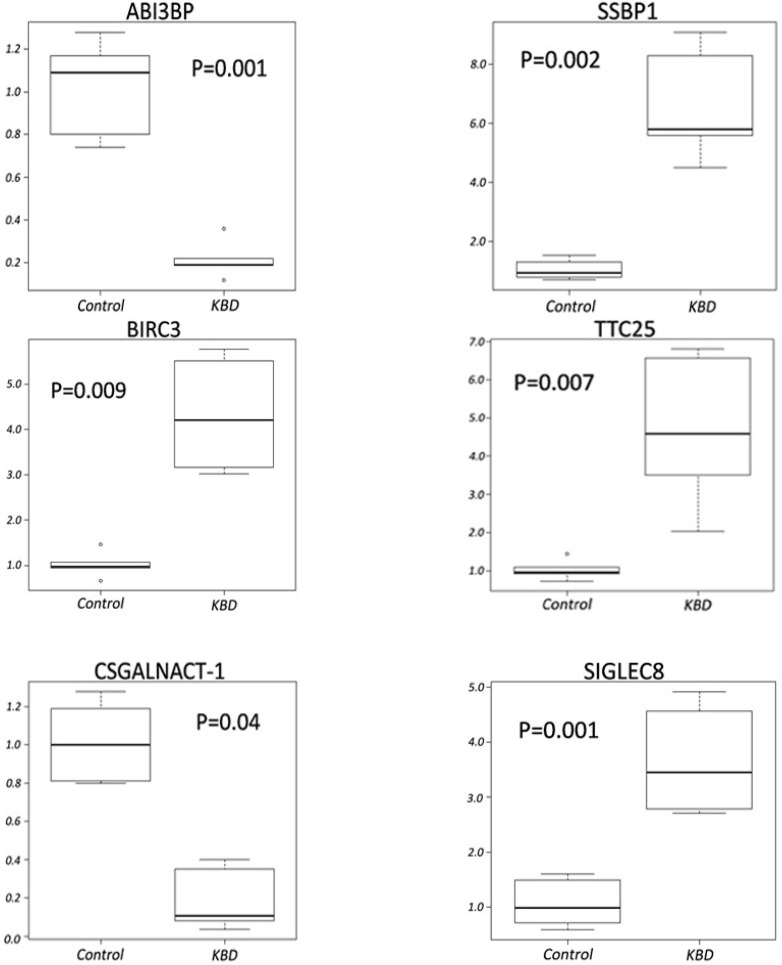
mRNA levels for ABI3BP, SSBP1, BIRC3, TTC25, CSGALNACT-1 and SIGLEC8 in PBMCs of controls and KBD patients.Steady-state mRNA levels were quantitated by two-step SYBR Green real time RT-PCR. The comparative Ct method was used for the calculation. The lines inside the boxes denote the medians. The boxes mark the interval between the 25th and 75th percentiles. The whiskers denote the interval between the 10th and 90th percentiles. The “○”indicates data outside the 10th and 90th percentiles.

To validate the microarray data, six differentially expressed genes were selected for qRT-PCR analysis using PBMCs samples from an additional 10 KBD patients. The expression levels of ABI3BP, BIRC3, CSGALNACT1, SIGLEC8, SSBP1, and TTC25 in the PBMCs of KBD patients were shown to be significantly different from the normal controls (Figure 1). Importantly, the changes were consistent with those revealed by the microarray data.

## 3. Discussion

Microarray analysis was used to identify the gene expression signatures of peripheral blood, which is a technique that has been demonstrated as an effective tool for a number of diseases, including schizophrenia and bipolar disorder [13], coronary artery disease [6], rheumatoid arthritis, juvenile arthritis, spondyloarthropathy [14,15], and osteoarthritis [7]. In this study, we identified a 20-gene signature in peripheral blood samples that accurately discriminates patients with KBD from the controls, as well as the degree of severity of KBD in patients. Our results suggest that these biomarkers may be used as a new method to diagnose early-stage KBD.

According to the national diagnostic criteria of Kashin-Beck disease (WS/T207-2010) in China, the diagnosis and determination of the severity of KBD is primarily based on the symptoms and metaphysis changes observed radiologically. However, KBD is an insidious disease with slow progression, and there are still suspected cases in endemic areas that have not manifested any pathological metaphyseal changes still have the risk of suffering from KBD. (WS/T207-2010) criteria tend to identify advanced KBD cases, but it could not identify patients at early stages. This indicates that the X-ray diagnosis method is much less sensitive for detecting the potential KBD or early stage patients. Therefore, we further emphasize the need for a non-invasive and sensitive early KBD diagnosis. Our results provided a high-performing 20-gene signature, which could be used as a biomarker to discriminate between patients with KBD and the normal controls with 90% accuracy, 85% sensitivity, and 95% specificity. In our test set (*n =* 40), two samples were diagnosed as KBD by application of the 20-gene signature, while they were diagnosed as normal by using (WS/T207-2010) criteria. Next, we are planning to validate the 20-gene signature in a large sample size of children with KBD as a biomarker diagnostic method to further establish a reliable and stable method of early KBD diagnosis.

The genes identified in this study belong to various functional categories, including metabolism, transcription, ion channel, signal transduction, transport proteins, apoptosis, immunity, growth factors, and receptors, among others. CACNG6 encodes a gamma subunit of a calcium channel, which functions in calcium ion transport across cell membranes. Calcium channels play an important role in the process of osteoblast differentiation into osteocytes and osteoclasts, and both calcium ions and calcium channels are crucial for apoptosis [16]. A number of hormones and cytokines that act directly on osteoblasts are regulated by calcium ions (via calcium channels) [17]. A previous study has shown the relationship between CACNG6 and KBD [18]. We found that CACNG6 was down-regulated in the peripheral blood of the KBD patients, which may reflect the differentiation disorder and apoptosis of chondrocytes observed in KBD patients. BIRC3 is a member of the BIRC family, and apoptosis regulation is one of the important functions of their encoded proteins [19,20]. Furthermore, BIRC2, BIRC3 and TRAF2 had been identified as critical genes in the activation of NF-κB signaling in TNF pathways [21]. At the same time, BIRC3 was observed to be significantly more up-regulated in KBD than control, which means BIRC3 might be one of the critical genes in NF-κB signal changing.

Growth differentiation factor 5 (GDF5), which plays an essential role in skeletal development, is a member of the bone morphogenetic (BMP) gene family and TGF-beta superfamily [22]. It also promotes the development, maintenance, and repair of synovial joint tissues, particularly for bone and cartilage [23]. It has been reported to be associated with OA in the Asian population [24]. A previous study examining the association between GDF5 gene polymorphisms and Kashin-Beck disease found that the polymorphism of haplotype TGC is associated with KBD [25]. In this study, the observed down-regulation of GDF5 indicates it plays an important role in the pathogenesis of KBD.

Among the 20 candidate genes, ABI3BP, CTSC, FZD1, ERH, CSGALNACT1, and SSBP1 were the most highly differentially expressed genes in the cartilage tissue of KBD or OA patients [1,12]. Cathepsin C (CTSC) is a lysosomal amino peptidase and a member of the papain family of cysteine proteinases. Cathepsins are synthesized in the endoplasmic reticulum as pre-proproteins consisting of a signal peptide, and can degrade extracellular matrix proteins, such as collagens [26]. Previous studies have demonstrated that enhancer of rudimentary homolog (ERH) may be a critical transcription regulator that also functions in the control of cell-cycle progression [27]. Single-stranded DNA binding protein 1, mitochondrial (SSBP1) is a housekeeping gene whose expression is regulated in response to signaling pathway that is involved in mitochondrial biogenesis in mammalian cells [28]. It is also a subunit of a single-stranded DNA (ssDNA)-binding complex involved in the maintenance of genome stability [29]. SSBP1 was up-regulated in our study, and KBD patients do exist with mitochondria dysfunction. The reactive oxygen content is increased in articular chondrocytes, and cytochrome c is released from mitochondria to cytoplasm which stimulates apoptosis [30].

Chondroitin sulfate *N*-acetylgalactosaminyl-transferase-1 (CSGALNACT-1) is one of the enzymes involved in chondroitin sulfate metabolism, which participates in chondroitin sulphate (CS) chain formation of aggrecan [12,31]. It also plays a key role in degeneration of the extracellular matrix of cartilage and in chondrogenesis at early developmental stages [32]. Additionally, some animal model research suggests that CSGALNACT-1 is important to endochondral ossification [33]. Down-regulation of this gene has been identified in the cartilage of KBD and OA patients, and it may also participate in the Wnt/β-catenin signaling pathway [14,34]. The same results were found in peripheral blood. These results together indicate that CSGALNACT-1gene expression is likely similar in cartilage and in PBMCs of KBD patients.

Protein products of frizzled genes are cell membrane receptors for Wnt proteins, which play multiple roles during development [35]. Furthermore, the Wnt/β-catenin signaling pathway is vital for development, growth, and homeostasis of the joints and the skeleton [36]. This study found that the gene FZD1 was down-regulated in the peripheral blood of KBD patients, while both FZD1 and FZD10 were up-regulated in the cartilage of KBD patients [12]. Although the expression was inconsistent, the alteration of FZD1 may contribute to the development of KBD through the Wnt/β-catenin signaling pathway. FZD1 and the Wnt/β-catenin signaling pathway could be the next targets in KBD studies. These genes may be worthy of further study regarding how alteration of cartilage in KBD influences changes in blood on a molecular level.

Microarrays have been used to predict and differentiate between benign and malignant tumors, as well as the size and degree of tumors, using gene expression ratios [37,38,39]. Using this technology, the identified gene signatures were able to accurately discriminate KBD patients from the controls. In this study, we reclassified the degree of severity of KBD using expression ratios of 20 candidate genes via discrimination analysis. The KBD patients could be accurately identified on a molecular level. Microarray analysis can thus provide greater information in cases in which it is difficult to classify or distinguish between the clinical types of KBD. The additional evidence that these 20 genes may be considered early diagnostic biomarkers of KBD is that the expression of candidate genes was not altered by age according to linear correlation analyses. Furthermore, our group performed another microarray experiment in PBMCs of juvenile KBD using the 20 signature genes. It was demonstrated that these 20 genes are also differentially expressed in PBMCs of children with KBD [40].

KBD and primary OA display similar clinical manifestations and pathologic changes in the articular cartilage [1]. However, KBD occurs mostly in children ages 3–12 years and becomes symptomatic in adults [41], Osteoarthritis, an age-related disease of the joints, is the most common form of arthritis, and is a leading cause of the disability in an aging western population [42,43]. Therefore, it is important to study the two types of osteoarthropathy comparatively. Previously, Duan *et al.* found a number of differentially expressed genes in the comparative analysis of gene expression profiles between KBD and OA in cartilage [1]. Marshall *et al*. found nine blood-based biomarkers, which could accurately discriminate the mild OA patients from normal controls [7], and were completely different from our 20-gene signature. Therefore, the 20-gene signature we identified might be considered a method of differential diagnosis between KBD and OA at early stages.

## 4. Experimental Section

### 4.1. Ethics Statement

This study was approved by the Institutional Review Board (IRB) of Xi’an Jiaotong University (Xi’an, China). All the participants gave their informed consent by signing a document that had been carefully reviewed by the IRB of Xi’an Jiaotong University.

### 4.2. Patients and Study Design

All subjects were randomly chosen from Yong-shou and Lin-you counties in Shaanxi province, which are the endemic areas of KBD, with a prevalence of 20.4% and 10.5%, respectively. Patients were diagnosed according to the national diagnostic criteria of Kashin-Beck disease (WS/T207-2010) in China (Table 3). The KBD patients were classified as having a first-degree (I) KBD manifestation due to multiple and symmetrical enlargements of phalanges or other joints, limitation of motion, arthralgia, mild muscle atrophy, and thickening of other limb joints. Patients with the symptoms of first degree KBD with additional brachydactylia were diagnosed as second-degree (II) KBD. The KBD patients were compared with age and sex-matched healthy controls without symptomatic and clinical KBD according to the criteria of (WS/T207-2010). All of patients and controls with other types of osteoarthropathy and other chronic diseases, such as cardiovascular disease, diabetes and hypertension, were excluded.

**Table 3:** Characteristics of the patients with KBD and the controls.

Variable	Microarray		Signature Identification			
	KBD	Control	Training Set		Test Set	
	(*N =* 100)	(*N =* 100)	KBD	Control	KBD	Control
			(*N =* 80)	(*N =* 80)	(*N =* 20)	(*N =* 20)
Age, year						
mean	57.91	53.43	57.50	53.20	56.20	52.34
range	43–79	40–77	43–79	40–77	43–75	46–72
Gender, N (%)						
Male	44 (44.0)	44 (44.0)	37 (46.2)	35 (43.7)	7 (35.0)	9 (45.0)
Female	56 (56.0)	56 (56.0)	43 (53.8)	45 (56.3)	13 (65.0)	11 (55.0)
KBD Degree, N (%)						
Degree I	50 (50.0)	-	38 (47.5)	-	12 (60.0)	-
Degree II	50 (50.0)		42 (52.5)		8 (40.0)	
Clinical Sign, N (%)						
EP ^a^	95 (95.0%)		77 (96.2%)		18 (90.0%)	
Brachydactylia	53 (53.0%)		45 (56.2%)		8 (40.0%)	

^a^ EP means enlargements of phalanges.

According to the inclusion and exclusion criteria of the sample selection described above, 100 patients (50 degree I and 50 degree II KBD patients) and 100 healthy controls were chosen for this study. These samples were divided into 100 pairs, each consisting of one KBD patients and one healthy control person, who was age and sex-matched. All subjects were of Chinese Han lineage. The microarray analyses were performed for each of the 100 pairs of the samples. Then, the 100 KBD patients and 100 healthy controls were completely randomized and divided into training and test sets. The training set consisted of 80 patients with KBD and 80 controls. In the test set, an independent cohort of 20 patients with KBD and 20 controls was used. The microarray analyses of the 100 paired samples were used to evaluate the expression level of 169 target genes. Then, the expression levels of target genes were used to establish a statistical model based on the SVM algorithm to predict KBD and healthy condition. The statistical model established in the training set was applied to the test set to validate the ability of the gene signature to identify the KBD patients.

**Figure 2:**
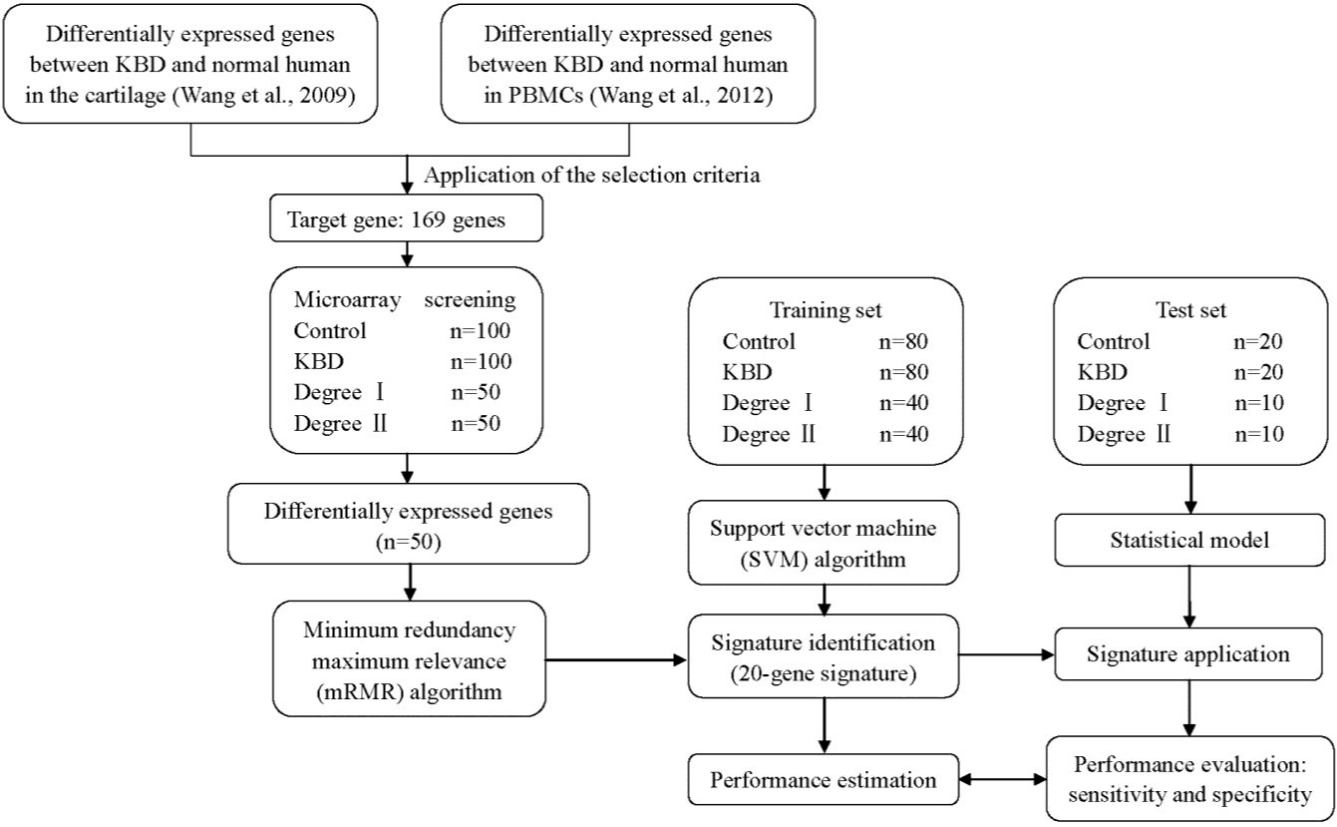
Study design. The gene expression in the peripheral blood of 100 patients with KBD and 100 controls were investigated in different parts. We first used previously published experiment data on gene expression profiles of adult articular cartilage and PBMCs with KBD to select the target genes that were significantly differentially expressed between patients with KBD and controls. One hundred pairs of microarray were then applied to evaluate the expression of the target genes to identify the differentially expressed genes based on a large population. A gene expression signature was identified using a training set (*n =* 160) and validated using an independent test set (*n =* 40).

An independent cohort of 5 patients with KBD and 5 controls (Table 4) was used to evaluate the validity of the obtained microarray data through parallel analysis of selected transcripts using a quantitative real-time reverse transcription polymerase chain reaction (qRT-PCR). Figure 2 depicts the different components of the study design, and Table 3 and Table 4 summarize the clinical characteristics of the samples in the study.

**Table 4:** Characteristics of the patients with KBD and the controls used for quantitative RT-PCR analysis.

Variable	KBD				Normal			
	*n*	Age (Years)	Male	Female	*n*	Age (Years)	Male	Female
1	1	69	1	0	1	65	1	0
2	1	62	1	0	1	56	1	0
3	1	57	1	0	1	56	1	0
4	1	58	0	1	1	55	0	1
5	1	52	0	1	1	48	0	1
Total	5	59.6	3	2	5	56.0	3	2

### 4.3. Selection of the Target Genes

In this study, 169 genes to be used in a custom-made microarray were selected as target genes from 2 databases of previously published microarray analyses [9,12]. In those studies, genes with a >2 or <0.5-fold-change in gene expression level obtained from KBD and healthy persons and a *p* value <0.05 and biologic function related to cartilage or selenium were selected. Finally, the 169 genes refined by application of selection criteria above were selected as target genes (The list of 169 target genes can be found in the online supplementary materials).

### 4.4. Blood Collection and the PBMC Isolation

Peripheral blood (4 mL) from each subject was collected into heparinized Vacutainer^®^ tubes (Becton Dickenson, San Jose, CA, USA) for the gene expression analysis. Leukocyte cell numbers were determined using a Hemovet 950 (Drew Scientific, Oxford, CT, USA). The PBMCs were separated from plasma by centrifuging the blood at 1500× *g* for 20 min. The cell pellets were resuspended in Hanks’ balanced salt solution (Gibco BRL/Invitrogen, Carlsbad, CA, USA). The cell suspensions were layered over 5 mL of Lympholyte-H (Cedarlane Labs, Hornby, BC, Canada) in a 15-mL Falcon tube and were centrifuged at 1500× *g* for 40 min. After rinsing twice with cold Hanks’ balanced salt solution, the cells were stored in RNAlater^®^ (Ambion Inc., Austin, TX, USA) until RNA isolation.

### 4.5. RNA Preparation

Total RNA was isolated from the PBMCs using Trizol^®^ reagent (Life Technologies Inc., Carlsbad, CA, USA) following the manufacturer’s recommended protocol. The quality and integrity of the extracted total RNA were determined using a high-resolution electrophoresis system (Agilent 2100 Bioanalyzer, Agilent Technologies, Palo Alto, CA, USA).

### 4.6. Microarray Hybridization

The isolated total RNA from all KBD patients and healthy controls were transcribed into complementary DNA (cDNA) and were then reverse-transcribed into cRNA and labeled with CyDye using the Amino Allyl Message Amp™ a RNA Amplification Kit (Ambion) according to the manufacturer’s instructions. Before reverse transcription, RNase-free DNase I was used to remove residual genomic DNA from the total RNA. For each sample, 0.5 µg of labeled cRNA was purified separately and then mixed with hybridization buffer before applying to microarrays. The custom-made primer array, which contains 169 oligonucleotide probes representing the selected 169 human genes (manufactured by the National Engineering Research Center for Miniaturized Detection System in Xi’an, China), was used to perform microarray hybridization following the recommended protocol. This is a two-color microarray that uses a two-channel labeling system. The control cRNA was labeled with Cy3, and the KBD cRNA was labeled with Cy5. The fluorescent spots that failed to pass the quality control procedure were not used for further analysis.

### 4.7. Gene Expression Analysis

Microarray slides were scanned with Gene-Pix 4000B (Axon Instruments Inc., Foster City, CA, USA), and GenePix Pro 3.0 software (Axon Instruments Inc.) was used to analyze the 16-bit TIFF images produced by the scanner. The fluorescent signal intensity was considered gene expression data. Ratio images for all of the spots were defined by accessing the gene list file describing the microarray location of each gene. After the average signal intensity was subtracted from the median back intensity, the gene expression data were imported into Excel spreadsheets (Microsoft Corp., Redmond, WA, USA) using Unigene and GenBank descriptors. A global normalization was conducted to calculate the relative expression levels between two samples using all of the detected genes. Each gene was identified as up-regulated or down-regulated if the fold change was more than two-fold or less than 0.5-fold, respectively. The fold change value is the ratio of the signal intensity of the KBD sample compared to that of the control sample in each pair. Significant differences in gene expression between the KBD and the control samples were determined by Student’s *t*-test. *p*-values were calculated by the standard combinatorial approach and then adjusted for multiple testing by the Bonferroni method. *p*-values less than 0.05 were considered statistically significant. All data have been deposited in NCBI GEO and are accessible through GEO series accession No. GSE59446 (http://www.ncbi.nlm.nih.gov/geo/query/acc.cgi?acc=GSE59446).

### 4.8. Statistical Analysis

The minimum redundancy maximum relevance (mRMR) algorithm was used for gene selection [44]. A library for support vector machine (LIBSVM) algorithm, a linear classification, was used for establishing a statistical model and classification [45], and leave-one-out cross validation (LOOCV) was conducted for the sampling. LOOCV was applied in this analysis. The Statistical Package for the Social Science for Windows version 18.0 (SPSS 18.0, IBM, Armonk, NY, USA) was applied to identify whether a correlation exists between age and gene expression by a linear correlation and ANOVA test. The Bayes discriminant analysis (BDA) algorithm was used to evaluate the accuracy of the KBD degree (I, II) identification with the expression ratio of the gene signature by using the SPSS 18.0 program. For the statistical analyses of quantitative real-time RT-PCR (qRT-PCR), the Mann-Whitney Wilcoxon Test was performed to determine the significance of level-of-expression differences for the selected genes between KBD and healthy controls.

### 4.9. Quantitative Real-Time PCR Validation

Quantitative real-time PCR was used to confirm the expression levels of 6 genes, which were the 4 most significantly up-regulated and the 2 most significantly down-regulated. Total RNA was isolated from an additional 10 subjects and prepared as for oligonucleotide microarray analysis, and then qRT-PCR was performed. The RNA was then converted into complementary DNA (cDNA) using Superscript II reverse transcriptase (Invitrogen, Karlsruhe, Germany) and random primers. QRT-PCR was performed using the ABI7500 Real-Time PCR system (Applied Biosystems, Foster City, CA, USA) according to the manufacturer’s instructions. The primer sequences are listed in Table 5. All primer and probe sets (ABI3BP, NM_015429.3; BIRC3, NM_001165.4; SSBP1, NM_001256510.1; SIGLEC8, NM_014442.2; TTC25, NM_031421.2; and CSGALNACT1, NM_001130518.1) were supplied by TaqMan^®^ Gene Expression Assays (Applied Biosystems). The relative fold-change for each individual gene was calculated by the comparative threshold cycle (*C*_t_) equation as follows: 2^−∆∆*C*t^, where ∆∆*C*_t_ = mean ∆*C*_t_ (KBD sample) − mean ∆*C*_t_ (control sample); ∆*C*_t_ = *C*_t_ (target gene) − *C*_t_ (housekeeping gene), where *C*_t_ values of target genes were normalized to *C*_t_ values of the housekeeping gene (β-ACTIN).

**Table 5:** Primers used in qRT-PCR validation of the microarray data.

Symbol	5' Primer Sequence	3' Primer Sequence	Amplicon Size (bp)
ABI3BP	GAAGATCACTGCCAGTTTGTGGA	CCTGGCGAACTGCTCTGAAATA	109
BIRC3	GACTCAGGTGTTGGGAATCTGGA	TGAGGGTAACTGGCTTGAACTTGAC	127
CSGALNACT1	CAGCTCTTGCTGCTGCTGTG	AAGGATGATCTTGCAGGCAGAA	139
SIGLEC8	CAGGTGTGACCACGACCAGTA	ACTGGCCCTCAAGGACTGAA	141
SSBP1	CCTCATCAGATGTGCAGGAATGTT	TGACCCACTCGCCCAAGTAAG	190
TTC25	AGATCGGCCGCTGCTACTTG	CCACCAGAACACTGGCATTCA	126
β-ACTIN	CGGAGTCAACGGATTTGGTCGTAT	AGCCTTCTCCATGGTGGTGAAGAC	120

## 5. Conclusions

This study describes the development of a blood-based, 20-gene signature that differentiates patients with KBD from controls with a high degree of accuracy in a large number of participants. These results provide a basis for further development of blood-based gene expression biomarkers for the detection of KBD. This first step is vital for identifying molecular markers for the early diagnosis of KBD.

## Supplementary Materials

Supplementary materials can be found at http://www.mdpi.com/1422-0067/16/05/11465/s1.

## References

[1] Duan C., Guo X., Zhang X.D., Yu H.J., Yan H., Gao Y., Ma W.J., Gao Z.Q., Xu P., Lammi M. (2010). Comparative analysis of gene expression profiles between primary knee osteoarthritis and an osteoarthritis endemic to Northwestern China, Kashin-Beck disease. Arthritis Rheumatol..

[2] Fang H., Guo X., Farooq U., Xia C., Dong R. (2012). Development and validation of a quality of life instrument for Kashin-Beck disease: An endemic osteoarthritis in China. Osteoarthr. Cartil..

[3] Zou K., Liu G., Wu T., Du L. (2009). Selenium for preventing Kashin-Beck osteoarthropathy in children: A meta-analysis. Osteoarthr. Cartil..

[4] Chasseur C., Suetens C., Nolard N., Begaux F., Haubruge E. (1997). Fungal contamination in barley and Kashin-Beck disease in Tibet. Lancet.

[5] Guo X., Ma W.J., Zhang F., Ren F.L., Qu C.J., Lammi M.J. (2014). Recent advances in the research of an endemic osteochondropathy in China: Kashin-Beck disease. Osteoarthr. Cartil..

[6] Ma J., Liew C.C. (2003). Gene profiling identifies secreted protein transcripts from peripheral blood cells in coronary artery disease. J. Mol. Cell. Cardiol..

[7] Marshall K.W., Zhang H., Yager T.D., Nossova N., Dempsey A., Zheng R., Han M., Tang H., Chao S., Liew C.C. (2005). Blood-based biomarkers for detecting mild osteoarthritis in the human knee. Osteoarthr. Cartil..

[8] Zhang F., Guo X., Wang W., Yan H., Li C. (2011). Genome-wide gene expression analysis suggests an important role of hypoxia in the pathogenesis of endemic osteochondropathy Kashin-Beck disease. PLoS ONE.

[9] Wang S., Guo X., Wu X.M., Lammi M.J. (2012). Genome-wide gene expression analysis suggests an important role of suppressed immunity in pathogenesis of Kashin-Beck disease. PLoS ONE.

[10] Murakami Y., Toyoda H., Tanahashi T., Tanaka J., Kumada T., Yoshioka Y., Kosaka N., Ochiya T., Taguchi Y.H. (2012). Comprehensive miRNA expression analysis in peripheral blood can diagnose liver disease. PLoS ONE.

[11] Ishimura M., Yamamoto H., Mizuno Y., Takada H., Goto M., Doi T., Hoshina T., Ohga S., Ohshima K., Hara T. (2013). A non-invasive diagnosis of histiocytic necrotizing lymphadenitis by means of gene expression profile analysis of peripheral blood mononuclear cells. J. Clin. Immunol..

[12] Wang W.Z., Guo X., Duan C., Ma W.J., Zhang Y.G., Xu P., Gao Z.Q., Wang Z.F., Yan H., Zhang Y.F. (2009). Comparative analysis of gene expression profiles between the normal human cartilage and the one with endemic osteoarthritis. Osteoarthr. Cartil..

[13] Tsuang M.T., Nossova N., Yager T., Tsuang M.M., Guo S.C., Shyu K.G., Glatt S.J., Liew C.C. (2005). Assessing the validity of blood-based gene expression profiles for the classification of schizophrenia and bipolar disorder: A preliminary report. Am. J. Med. Genet. B Neuropsychiatr. Genet..

[14] Barnes M.G., Aronow B.J., Luyrink L.K., Moroldo M.B., Pavlidis P., Passo M.H., Grom A.A., Hirsch R., Giannini E.H., Colbert R.A. (2004). Gene expression in juvenile arthritis and spondyloarthropathy: Pro-angiogenic ELR^+^ chemokine genes relate to course of arthritis. Rheumatology.

[15] Niimoto T., Nakasa T., Ishikawa M., Okuhara A., Izumi B., Deie M., Suzuki O., Adachi N., Ochi M. (2010). MicroRNA-146a expresses in interleukin-17 producing T cells in rheumatoid arthritis patients. BMC Musculoskelet. Disord..

[16] Gu G.S., Che M.X. (2009). Calcium channel of osteoblast. Chin. J. Clin. Oncol. Rehabil..

[17] Guggino S.E., Wagner J.A., Snowman A.M., Hester L.D., Sacktor B., Snyder S.H. (1988). Phenylalkylamine-sensitive calcium channels in osteoblast-like osteosarcoma cells. Characterization by ligand binding and single channel recordings. J. Biol. Chem..

[18] Wang X., Wang S., He S., Zhang F., Tan W., Lei Y., Yu H., Li Z., Ning Y., Xiang Y. (2013). Comparing gene expression profiles of Kashin-Beck and Keshan diseases occurring within the same endemic areas of China. Sci. China Life Sci..

[19] Gyrd-Hansen M., Meier P. (2010). IAPs: From caspase inhibitors to modulators of NF-κB, inflammation and cancer. Nat. Rev. Cancer.

[20] Tan B.M., Zammit N.W., Yam A.O., Slattery R., Walters S.N., Malle E., Grey S.T. (2013). Baculoviral inhibitors of apoptosis repeat containing (BIRC) proteins fine-tune TNF-induced nuclear factor κB and c-Jun *N*-terminal kinase signalling in mouse pancreatic beta cells. Diabetologia.

[21] Rothe M., Pan M.G., Henzel W.J., Ayres T.M., Goeddel D.V. (1995). The TNFR2-TRAF signaling complex contains two novel proteins related to baculoviral-inhibitor of apoptosis proteins. Cell.

[22] Chang S.C., Hoang B., Thomas J.T., Vukicevic S., Luyten F.P., Ryba N.J., Kozak C.A., Reddi A.H., Moos M. (1994). Cartilage-derived morphogenetic proteins. New members of the transforming growth factor-β superfamily predominantly expressed in long bones during human embryonic development. J. Biol. Chem..

[23] Mikic B. (2004). Multiple effects of GDF-5 deficiency on skeletal tissues: Implications for therapeutic bioengineering. Ann. Biomed. Eng..

[24] Miyamoto Y., Mabuchi A., Shi D., Kubo T., Takatori Y., Saito S., Fujioka M., Sudo A., Uchida A., Yamamoto S. (2007). A functional polymorphism in the 5' UTR of GDF5 is associated with susceptibility to osteoarthritis. Nat. Genet..

[25] Gao X., Zhao Y., Yu M., Tu Q., Wu L., Lai J., Guo X. (2010). Association between GDF5 gene polymorphism and Kashin-Beck disease. J. Xi’an Jiaotong Univ. (Med. Sci.).

[26] Wolters P.J., Chapman H.A. (2000). Importance of lysosomal cysteine proteases in lung disease. Respir. Res..

[27] Jin T., Guo F., Serebriiskii I.G., Howard A., Zhang Y.Z. (2007). A 1.55 Å resolution X-ray crystal structure of HEF2/ERH and insights into its transcriptional and cell-cycle interaction networks. Proteins.

[28] Tiranti V., Rossi E., Ruiz-Carrillo A., Rossi G., Rocchi M., DiDonato S., Zuffardi O., Zeviani M. (1995). Chromosomal localization of mitochondrial transcription factor A (TCF6), single-stranded DNA-binding protein (SSBP), and endonuclease G (ENDOG), three human housekeeping genes involved in mitochondrial biogenesis. Genomics.

[29] Huang J., Gong Z.H., Ghosal G., Chen J.J. (2009). SOSS complexes participate in the maintenance of genomic stability. Mol. Cell.

[30] Liu J.T., Guo X., Ma W.J., Zhang Y.G., Xu P., Yao J.F., Bai Y.D. (2010). Mitochondrial function is altered in articular chondrocytes of an endemic osteoarthritis, Kashin-Beck disease. Osteoarthr. Cartil..

[31] Gulberti S., Jacquinet J.C., Chabel M., Ramalanjaona N., Magdalou J., Netter P., Coughtrie M.W., Ouzzine M., Fournel-Gigleux S. (2012). Chondroitin sulfate *N*-acetylgalactosaminyltransferase-1 (CSGalNAcT-1) involved in chondroitin sulfate initiation: Impact of sulfation on activity and specificity. Glycobiology.

[32] Sakai K., Kimata K., Sato T., Gotoh M., Narimatsu H., Shinomiya K., Watanabe H. (2007). Chondroitin sulfate *N*-acetylgalactosaminyltransferase-1 plays a critical role in chondroitin sulfate synthesis in cartilage. J. Biol. Chem..

[33] Sato T., Kudo T., Ikehara Y., Ogawa H., Hirano T., Kiyohara K., Hagiwara K., Togayachi A., Ema M., Takahashi S. (2011). Chondroitin sulfate *N*-acetylgalactosaminyltransferase 1 is necessary for normal endochondral ossification and aggrecan metabolism. J. Biol. Chem..

[34] Zheng J., Wu C., Ma W., Zhang Y., Hou T., Xu H., Wu S., Yao X., Guo X. (2013). Abnormal expression of chondroitin sulphate *N*-acetylgalactosaminyltransferase 1 and Hapln-1 in cartilage with Kashin-Beck disease and primary osteoarthritis. Int. Orthop..

[35] Xu L., Tan L., Goldring M.B., Olsen B.R., Li Y. (2001). Expression of frizzled genes in mouse costochondral chondrocytes. Matrix Biol..

[36] Lories R.J., Corr M., Lane N.E. (2013). To Wnt or not to Wnt: The bone and joint health dilemma. Nat. Rev. Rheumatol..

[37] Ma Y., Qian Y., Wei L., Abraham J., Shi X., Castranova V., Harner E.J., Flynn D.C., Guo L. (2007). Population-based molecular prognosis of breast cancer by transcriptional profiling. Clin. Cancer Res..

[38] Prasad N.B., Somervell H., Tufano R.P., Dackiw A.P., Marohn M.R., Califano J.A., Wang Y., Westra W.H., Clark D.P., Umbricht C.B. (2008). Identification of genes differentially expressed in benign *versus versus* malignant thyroid tumors. Clin. Cancer Res..

[39] Xu Y., Xu Q., Yang L., Ye X., Liu F., Wu F., Ni S., Tan C., Cai G., Meng X. (2013). Identification and validation of a blood-based 18-gene expression signature in colorectal cancer. Clin. Cancer Res..

[40] Wen Y., Zhang F., Li C., He S., Tan W., Lei Y., Zhang Q., Yu H., Zheng J., Guo X. (2014). Gene expression analysis suggests bone development-related genes GDF5 and DIO2 are involved in the development of Kashin-Beck disease in children rather than adults. PLoS ONE.

[41] Hinsenkamp M. (2001). Kashin-Beck disease. Int. Orthop..

[42] Felson D.T., Lawrence R.C., Dieppe P.A., Hirsch R., Helmick C.G., Jordan J.M., Kington R.S., Lane N.E., Nevitt M.C., Zhang Y. (2000). Osteoarthritis: New insights. Part 1: The disease and its risk factors. Ann. Intern. Med..

[43] Hopwood B., Gronthos S., Kuliwaba J.S., Robey P.G., Findlay D.M., Fazzalari N.L. (2005). Identification of differentially expressed genes between osteoarthritic and normal trabecular bone from the intertrochanteric region of the proximal femur using cDNA microarray analysis. Bone.

[44] Ding C., Peng H. (2005). Minimum redundancy feature selection from microarray gene expression data. J. Bioinform. Comput. Biol..

[45] Chang C.C., Lin C.J. (2011). LIBSVM: A library for support vector machines. ACM Trans. Intell. Syst. Technol..

